# Ablação Baseada em Substrato de Fibrilação Ventricular Relacionada a Purkinje em um Paciente Idoso com Cardiomiopatia Isquêmica

**DOI:** 10.36660/abc.20220774

**Published:** 2023-09-19

**Authors:** Nurullah Çetin, Mustafa Özcan Soylu, Batuhan Özbaş, Özgür Bayturan, Uğur Kemal Tezcan

**Affiliations:** 1 Departamento de Cardiologia Manisa Celal Bayar University Faculty of Medicine Manisa Turquia Departamento de Cardiologia - Manisa Celal Bayar University, Faculty of Medicine, Manisa – Turquia

**Keywords:** Ablação por Cateter, Fibrilação Ventricular, Celulas de Purkinje, Cardiomiopatia Isquêmica, Idoso

## Introdução

As arritmias ventriculares fatais têm incidência aumentada no caso de doenças cardíacas estruturais, como a cardiomiopatia isquêmica.^1^ Sabe-se que as contrações ventriculares prematuras (CVPs) originárias das fibras de Purkinje podem induzir taquicardia ventricular polimórfica (TVPM) e, posteriormente, fibrilação ventricular (FV) em pacientes com cardiomiopatia isquêmica.^2,3^ As arritmias ventriculares associadas a Purkinje geralmente não respondem aos antiarrítmicos convencionais. A ablação por cateter é uma estratégia de tratamento favorável e salva-vidas para FV desencadeada por fibras de Purkinje.^4^

## Relato de Caso

Paciente do sexo masculino, 89 anos, submetido a angiografia coronária e implante de stent na artéria coronária descendente anterior (DAE) há dois anos, procurou nosso ambulatório com queixas recentes de dispneia crescente. Não houve queixas cardíacas adicionais, como dor torácica, palpitações e síncope. A ecocardiografia transtorácica mostrou que a fração de ejeção do ventrículo esquerdo diminuiu de 40% para 20% em comparação com dois anos atrás, com o ventrículo esquerdo severamente dilatado e acinesia do ápice, médio a apical, parede anterior e anteroseptal. O paciente, que também apresenta sintomas de insuficiência cardíaca classe II da NYHA, é internado em nossa unidade de terapia intensiva para exames complementares. O exame físico demonstrou crepitações bilaterais nos campos pulmonares basais. O eletrocardiograma (ECG) revelou que o ritmo era sinusal sem CVPs. Os valores laboratoriais, incluindo eletrólitos séricos, estavam dentro dos limites normais.

**Figura 1:**
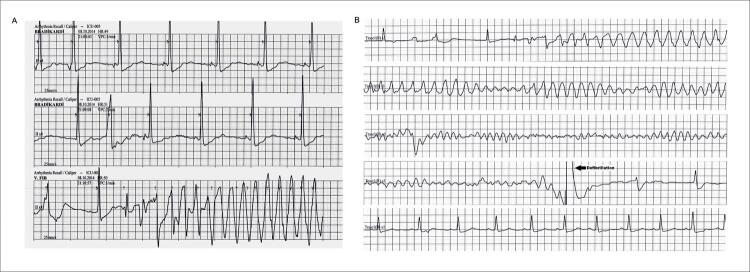
– Registro do monitor cardíaco de taquicardia ventricular polimórfica (TVPM)/fibrilação ventricular (FV). A) Início da TVPM. B) Registro longo do mesmo episódio. Degeneração de TVPM para FV e término por desfibrilação externa.

Estava em ritmo sinusal na admissão, mas depois teve uma parada cardíaca onde o monitoramento cardíaco demonstrou TVPM degenerado para FV na noite de sua hospitalização (Figura 1A). O ritmo sinusal foi restabelecido pela desfibrilação (Figura 1B). Isquemia coronariana foi considerada a causa mais provável de TVPM, sendo iniciada terapia anti-isquêmica e amiodarona endovenosa. No dia seguinte, o paciente foi submetido a coronariografia mostrando que o stent DAE estava patente, e não havia estenose significativa nos outros vasos em comparação com a angiografia mais recente feita há dois anos. Apesar da terapia médica máxima, o paciente apresentou quatro episódios de TV polimórfica degenerada em FV terminada por desfibrilação elétrica nos 3 dias seguintes. A partir daí, foi planejado procedimento de ablação por cateter de radiofrequência (RF).

**Figura 2:**
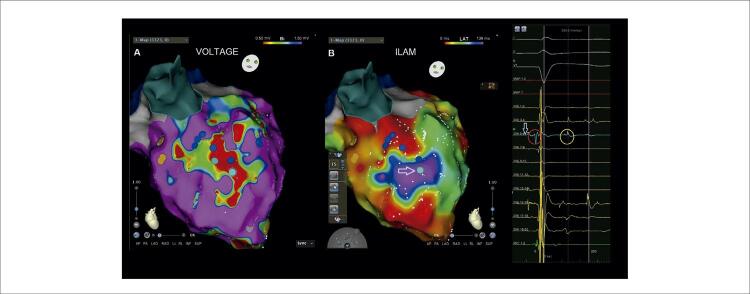
– Mapa CARTO eletroanatômico do ventrículo esquerdo do paciente. A) O mapeamento de voltagem durante o ritmo sinusal demonstra uma área cicatricial no meio do anterosepto. B) O mapeamento isocronal de ativação tardia (ILAM) mostra o agrupamento isocronal consistente com uma zona de desaceleração na mesma área. Marcas turquesa representam locais onde Purkinje e potenciais diastólicos tardios são registrados em cicatrizes densas e áreas lotadas isocrônicas. Purkinje no círculo vermelho e potenciais diastólicos tardios no círculo amarelo são registrados no eletrograma intracardíaco no ponto marcado com uma seta turquesa.

**Figura 3:**
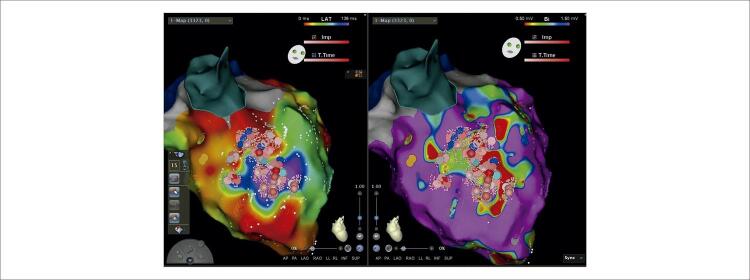
– Lesões de ablação entregues no anteroseptum médio em áreas de potenciais de Purkinje e potenciais diastólicos tardios adjuvantes para ZD e zona de borda de cicatriz.

O mapeamento anatômico foi realizado pelo cateter PentaRay (Biosense Webster) utilizando o sistema eletroanatômico (EAM) CARTO (Biosense Webster). Derivações eletrográficas de superfície e eletrogramas intracardíacos (EGMs) foram registrados continuamente. Um mapa de voltagem foi criado para identificar a zona cicatricial relacionada ao infarto e o miocárdio normal. O mapeamento de voltagem durante o ritmo sinusal demonstrou áreas cicatriciais no anterosepto médio-basal (Figura 2A). O mapeamento ısocronal de ativação tardia (ILAM) demonstrou uma área de aglomeração/zona de desaceleração (ZD) isocronal no anteroseptum basal médio adjacente à área da cicatriz (Figura 2B, Vídeo 1). ZD foram definidas como regiões com > 3 isócronas dentro de um raio de 1 cm. A lentidão extrema da condução foi definida como regiões de apinhamento isócrono com atividade fracionada local contínua dentro da ZD. No nosso caso, uma única área de ZD foi observada no anteroseptum basal médio. Como não havia morfologia direcionável, o ILAM foi realizado apenas em ritmo sinusal. Além disso, tanto o potencial de Purkinje quanto o diastólico tardio foram observados em EGMs nessa região. Não foi possível realizar a ativação e o mapeamento de marcapasso porque não havia CVP no ECG de superfície e o paciente não tolerava TVPM/FV. Portanto, identificamos ZD em ILAM como o alvo da ablação, com Purkinje e potenciais tardios em EGMs. A ablação foi realizada com sucesso na zona limítrofe da área de baixa voltagem/cicatriz no anteroseptum médio, especificamente na região de ZD, visando o Purkinje e os potenciais tardios. A ablação por RF foi realizada com um cateter de ablação de força de contato de curva D/F bidirecional de ponta irrigada (SmartTouch, Biosense Webster), visando força de contato média de 10g apontada para o miocárdio com 40 watts e fluxo de 30 mL/min. O tempo total de RF foi de 25 minutos e o tempo total do procedimento foi de 3 horas sem intercorrências. O paciente foi submetido à ablação do substrato anormal identificado por potenciais de baixa voltagem, fracionados e tardios. A zona da borda da cicatriz e os potenciais de Purkinje adjacentes a essa região também foram determinados como alvos de ablação (Figura 3). A energia de RF foi aplicada até que os potenciais fragmentado, tardio e de Purkinje desaparecessem. Após o procedimento de ablação, nenhuma arritmia ventricular sustentada foi induzida por estimulação extra tripla aplicada dos ventrículos direito e esquerdo após o procedimento de ablação. Um cardioversor desfibrilador implantável foi inserido no paciente dois dias após o procedimento. Durante os acompanhamentos do primeiro e terceiro mês, ele não experimentou nenhum ataque de TV-FV ou recebeu qualquer choque.

**Vídeo 1:**
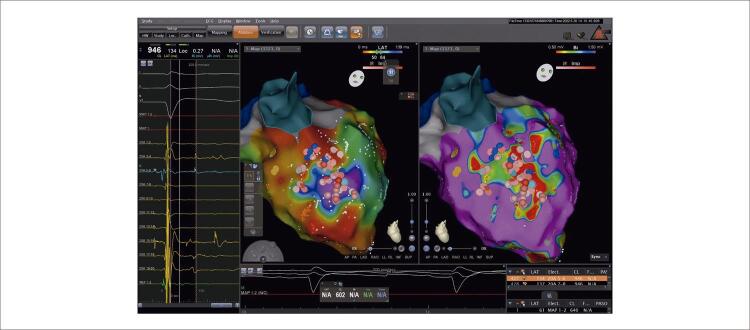
– O mapeamento de ativação tardia ısócrona mostra aglomeração isócrona consistente com baixa tensão/área de cicatriz.

Link: http://abccardiol.org/supplementary-material/2023/12009/2022-0774_RC_Video_1_1.mp4

## Discussão

A TVPM/FV pode ser desencadeada e mantida pela atividade originada do sistema de Purkinje distal localizado na zona limítrofe da cicatriz.^5^ Evidências emergentes em várias situações clínicas mostraram que os indutores predominantes de FV em pacientes são gatilhos originários do sistema de Purkinje distal.^6-8^ Prevê-se que a proximidade das células do tecido de Purkinje com o endocárdio permita o contato e a perfusão com o sangue cavitário, sobrevivendo assim ao infarto transmural em modelos experimentais. Essas fibras de Purkinje sobreviventes que cruzam a região limítrofe da cicatriz mostram alta automaticidade, atividade desencadeada e excitabilidade supernormal; isso, combinado com o prolongamento da

duração do potencial de ação nessa região, pode resultar no ambiente necessário para TVPM e FV.^9^ Ondas espirais reentrantes ou atividades focais sustentando FV podem ser fixadas em áreas de miocárdio anormal com fibras de Purkinje adjacentes.

A terapia médica com drogas antiarrítmicas, como amiodarona e cardioversor-desfibrilador implantável, continua sendo a base no manejo da FV. Em uma revisão recente de 86 pacientes com FV, a quinidina foi a mais eficaz entre as drogas administradas por via oral e muito melhor do que a amiodarona e a flecainida.^10^ No entanto, os dados sobre a eficácia dos antiarrítmicos no tratamento da FV relacionada a Purkinje são insuficientes. Portanto, a ablação por cateter após o mapeamento de alta resolução pode ser uma estratégia de tratamento primária e salvadora de pacientes selecionados com TVPM e FV.

O tratamento curativo concentra-se na ablação do batimento inicial de FV que corresponde à morfologia das CVPs anteriores. Haissaguerre et al.,^6^ relataram que até 85% das CVPs desencadeantes estavam localizadas no sistema de condução de Purkinje, e a ablação dos desencadeantes resultou em uma impressionante liberdade de FV (89%) sem drogas antiarrítmicas em dois anos de acompanhamento.^6^ A intolerabilidade do paciente ou a falta de monitoramento das CVPs durante o procedimento não permite o mapeamento de ativação ou arrastamento e invalida essa estratégia. O tratamento antiarrítmico, especialmente aplicado no período até a ablação, pode causar supressão das CVPs durante o procedimento. Em nosso paciente, o tratamento com amiodarona pode ter causado supressão de CVPs durante o procedimento. Nesta situação, a estratégia baseada em substrato pode ser uma alternativa.

A presença de um substrato local com atividade de Purkinje parece essencial para o aparecimento e manutenção da FV. Especialmente durante a fase inicial de FV, a maioria dos drivers se origina de substratos estruturais definidos eletrofisiologicamente.^11^ As heterogeneidades estruturais são críticas para a ocorrência de reentradas, diminuindo as velocidades de condução e, assim, ancorando as reentradas. Foi demonstrado anteriormente que, especialmente para TV reentrante, as regiões críticas estão localizadas nas regiões de ativação lenta ou em ZD durante o ritmo sinusal.^12^ A delineação do substrato com base no mapeamento de voltagem bipolar é convencionalmente usada para orientar estratégias de ablação direcionadas a regiões de baixa voltagem, mas as áreas de cicatriz variam dependendo das técnicas de registro. Por outro lado, nem todas as regiões cicatriciais apresentam o mesmo potencial de arritmogenicidade. No entanto, há evidências de que as regiões de desaceleração detectadas durante o ritmo sinusal são altamente arritmogênicas e atuam como um nicho para reentrada.^13^

Mapear o substrato com voltagem pode ajudar a identificar locais de cicatriz que podem participar da reentrada. Além disso, o ILAM visa identificar áreas de ativação lenta ou atrasada ou ZD durante o ritmo sinusal. Aziz et al. realizaram ablação em ZD visando priorizar regiões ativadas posteriormente com apinhamento isócrono máximo em pacientes com TV relacionada à cicatriz.^13^ Eles observaram que 63% das ZD estavam em cicatriz densa, 35% em tecido cicatricial misto e apenas 2% em zona de voltagem normal. A liberdade de TV foi de 80% no seguimento de um ano. Em nosso caso, observamos apenas uma ZD, e essa região era adjacente à zona de borda da cicatriz e continha Purkinje e potenciais tardios nos registros do EGM. Identificamos essa região como o alvo da ablação.

## Conclusão

Independentemente da idade, a ablação por cateter de RF é uma importante opção de tratamento para salvar vidas para FV. O ILAM pode desempenhar um papel fundamental para os alvos de ablação baseados em substrato em pacientes nos quais a FV não pode ser induzida, ou CPVs não são observados durante o procedimento.
