# Supplementary material for: Ablação Baseada em Substrato de Fibrilação Ventricular Relacionada a Purkinje em um Paciente Idoso com Cardiomiopatia Isquêmica
Source: Arq Bras Cardiol. 2023 Sep 19;120(9):e20220774. [Article in Portuguese] doi: 10.36660/abc.20220774 (PMC10519349; doi:10.36660/abc.20220774)
Supplement: Translation

# Translation: Substrate-Based Ablation of Purkinje-Related Ventricular Fibrillation in an Elderly Patient with Ischemic Cardiomyopathy

**Authors:** Nurullah Çetin, Mustafa Özcan Soylu, Batuhan Özbaş, Özgür Bayturan, Uğur Kemal Tezcan

**Affiliations:** 1 Turkey Departamento de Cardiologia - Manisa Celal Bayar University, Faculty of Medicine, Manisa – Turkey

**Keywords:** Catheter Ablation, Ventricular Fibrillation, Purkinje Cells, Myocardial Ischemia, Aged

## Introduction

Fatal ventricular arrhythmias have an increased incidence in the case of structural heart diseases such as ischemic cardiomyopathy.^1^ It is known that ventricular premature contractions (PVCs) originating from Purkinje fibers can induce polymorphic ventricular tachycardia (PMVT) and, subsequently, ventricular fibrillation (VF) in patients with ischemic cardiomyopathy.^2,3^ Ventricular arrhythmias associated with Purkinje usually do not respond to conventional antiarrhythmic drugs. Catheter ablation is a favorable and life-saving treatment strategy for VF triggered by Purkinje fibers.^4^

## Case Report

An 89-year-old male patient who underwent coronary angiography and left anterior descending coronary artery (LAD) stent implantation two years ago visited our outpatient clinic with recent complaints of increasing dyspnea. There were no additional cardiac complaints, such as chest pain, palpitations, and syncope. Transthoracic echocardiography showed left ventricular ejection fraction has decreased from 40% to 20% compared to two years ago, with the severely dilated left ventricle and akinesis of the apex, mid to apical, anterior wall, and anteroseptum. The patient, who also has NYHA class II heart failure symptoms, is admitted to our intensive care unit for further examination. Physical examination demonstrated bilateral crackles on basal lung fields. Electrocardiography (ECG) revealed that the rhythm was sinus without any PVCs. Laboratory values, including serum electrolytes, were within normal limits.

He was in sinus rhythm on admission but then had a cardiac arrest where cardiac monitoring demonstrated PMVT degenerated to VF on the evening of his hospitalization (Figure 1A). Sinus rhythm was restored by defibrillation (Figure 1B). Coronary ischemia was considered the most probable cause of PMVT, and anti-ischemic therapy and intravenous amiodarone were started. The following day, the patient underwent coronary angiography showing the LAD stent was patent, and there was no significant stenosis in the other vessels compared to the most recent angiography two years ago. Despite maximal medical therapy, the patient experienced four episodes of polymorphic VT degenerated to ventricular fibrillation terminated by electrical defibrillation within the following 3 days. Thereupon, a radiofrequency (RF) catheter ablation procedure was planned.

**Figure 1:** – Cardiac monitor recording of polymorphic ventricular tachycardia (PMVT)/ventricular fibrillation (VF). A) Onset of PMVT. B) Long record of the same episode. Degeneration of PMVT to VF and termination by external defibrillation.

Anatomical mapping was performed by PentaRay (Biosense Webster) catheter using the CARTO (Biosense Webster) electroanatomical (EAM) system. Surface electrographic leads and intracardiac electrograms (EGMs) were continuously recorded. A voltage map was created to identify infarct-related scar zone and normal myocardium. Voltage mapping during sinus rhythm demonstrated scar areas in the mid-basal anteroseptum (Figure 2A). Isochronal late activation mapping (ILAM) demonstrated an area of isochronal crowding/deceleration zone (DZ) on the mid-basal anteroseptum adjacent to the scar area (Figure 2B, Video 1). DZ were defined as regions with >3 isochrones within a 1cm radius. Extreme conduction slowing was defined as regions of isochronal crowding with continuous local fractionated activity within the DZ. In our case, a single area of DZ was noted at the mid-basal anteroseptum. Since there was no targetable morphology, ILAM was performed only in sinus rhythm. In addition, both Purkinje and late diastolic potentials were observed in EGMs in this region. We could not perform activation and pace mapping because there was no PVC in the surface ECG, and the patient could not tolerate PMVT/VF. Therefore, we identified DZ in ILAM as the ablation target, with both Purkinje and late potentials in EGMs. Ablation was successfully performed in the border zone of low voltage/scar area on the mid anteroseptum, specifically in the region of DZ, targeting the Purkinje and late potentials. The RF ablation was performed with an irrigated tip bidirectional D/F curve contact force ablation catheter (SmartTouch, Biosense Webster), aiming an average contact force of 10g pointed toward the myocardium with 40 watts and a 30 mL/min flow rate. The total RF time was 25 minutes, and the total procedure time was 3 hours without complications. The patient underwent ablation of the abnormal substrate as identified by low-voltage, fractionated, and late potentials. Scar border zone and Purkinje potentials adjacent to this region were also determined as ablation targets (Figure 3). RF energy was applied until the fragmented, late, and Purkinje potentials disappeared. After the ablation procedure, no sustained ventricular arrhythmia was induced by triple extra stimulation applied from the right and left ventricles after the ablation procedure. An implantable cardioverter defibrillator was inserted into the patient two days after the procedure. During the first and third month follow-ups, he did not experience any VT-VF attack or receive any shock.

**Figure 2:** – Electroanatomic CARTO map of the patient’s left ventricle. A) Voltage mapping during sinus rhythm demonstrates a scar area in the mid-anteroseptum. B) Isochronal late activation mapping (ILAM) shows isochronal crowding consistent with a deceleration zone in the same area. Turquoise tags represent sites where Purkinje and late diastolic potentials are recorded in both dense scar and isochronal crowded areas. Purkinje in the red circle and late diastolic potentials in the yellow circle are recorded in the intracardiac electrogram at the point marked with a turquoise arrow.

**Video 1:** – Isochronal Late Activation Mapping shows isochronal crowding consistent with low voltage /scar area.

Link: http://abccardiol.org/supplementary-material/2023/12009/2022-0774_RC_Video_1_1.mp4

**Figure 3:** – Ablation lesions delivered to the mid anteroseptum at areas of Purkinje potentials and late diastolic potentials adjuvant to DZ and scar border zone.

## Discussion

PMVT/VF could be triggered and maintained by activity originating from the distal Purkinje system localized to the border zone of scar.^5^ Evidence emerging in various clinical situations has shown that the predominant inducers of VF in patients are triggers originating from the distal Purkinje system.^6-8^ The proximity of Purkinje tissue cells to the endocardium is predicted to allow contact and perfusion with cavitary blood, thereby surviving transmural infarction in experimental models. These surviving Purkinje fibers crossing the border region of the scar show high automaticity, triggered activity, and supernormal excitability; this, combined with the prolongation of the action potential duration in this region, may result in the necessary environment for PMVT and VF.^9^ Reentrant spiral waves or focal activities sustaining VF may be fixed in areas of abnormal myocardium with adjacent Purkinje fibers.

Medical therapy with antiarrhythmic drugs such as amiodarone and implantable cardioverter-defibrillator remains the mainstay in managing VF. In a recent review of 86 patients with VF, quinidine was the most effective among the orally administered drugs and far better than amiodarone and flecainide.^10^ However, data on antiarrhythmic drugs’ efficacy in treating Purkinje-related VF are insufficient. Therefore, catheter ablation following high-resolution mapping may be a primary and life-saving treatment strategy for selected patients with PMVT and VF.

Curative treatment focuses on ablation of the initiating beat of VF that matches the preceding PVCs morphology. Haissaguerre et al.^6^ reported that up to 85% of trigger PVCs were localized to the Purkinje conduction system, and ablation of triggers resulted in impressive (89%) VF freedom without antiarrhythmic drugs at two-year follow-up.^6^ The patient’s intolerability or the lack of monitoring of PVCs during the procedure does not allow activation or entrainment mapping and invalidates this strategy. Antiarrhythmic treatment, especially applied during the period until ablation, may cause PVCs suppression during the procedure. In our patient, the amiodarone treatment might have caused suppression of PVCs during the procedure. In this situation, the substrate-based strategy may be an alternative.

The presence of a local substrate with Purkinje activity seems essential for the onset and maintenance of VF. Especially during the Initial VF phase, most drivers originate from electrophysiologically defined structural substrates.^11^ Structural heterogeneities are critical for the occurrence of reentries by decreasing the conduction velocities and thereby anchoring reentries. It has been previously shown that, especially for reentrant VT, the critical regions are localized to the slowing activation regions or at DZ during sinus rhythm.^12^ Substrate delineation based on bipolar voltage mapping is conventionally used to guide ablation strategies directed at low-voltage regions, but scar areas vary depending on recording techniques. On the other hand, not all scar regions harbor the same potential for arrhythmogenicity. However, there is evidence that deceleration regions detected during sinus rhythm are highly arrhythmogenic and act as a nidus for reentry.^13^

Mapping the substrate with voltage can help identify sites of scar that may participate in reentry. Additionally, ILAM aims to identify areas of slow or delayed activation or DZ during sinus rhythm. Aziz et al. performed ablation at DZ by targeting prioritizing later activated regions with maximal isochronal crowding in patients with scar-related VT.^13^ They observed that 63% of DZ were in a dense scar, 35% were in mixed scar tissue, and only 2% were in a normal voltage zone. VT freedom was 80% at the one-year follow-up. In our case, we observed only one DZ, and this region was adjacent to the scar border zone and contained Purkinje and late potentials in EGM recordings. We identified this region as the ablation target.

## Conclusion

Regardless of age, RF catheter ablation is an important life-saving treatment option for VF. ILAM may play a pivotal role for substrate-based ablation targets in patients in whom VF cannot be induced, or PVCs are not observed during the procedure.
